# Functional Imaging of Autonomic Regulation: Methods and Key Findings

**DOI:** 10.3389/fnins.2015.00513

**Published:** 2016-01-26

**Authors:** Paul M. Macey, Jennifer A. Ogren, Rajesh Kumar, Ronald M. Harper

**Affiliations:** ^1^UCLA School of Nursing, University of California at Los AngelesLos Angeles, CA, USA; ^2^Brain Research Institute, University of California at Los AngelesLos Angeles, CA, USA; ^3^Department of Neurobiology, University of California at Los AngelesLos Angeles, CA, USA; ^4^Department of Anesthesiology, University of California at Los AngelesLos Angeles, CA, USA; ^5^Department of Radiological Sciences, David Geffen School of Medicine at University of California at Los AngelesLos Angeles, CA, USA; ^6^Department of Bioengineering, University of California at Los AngelesLos Angeles, CA, USA

**Keywords:** cerebellum, fMRI, insula, parasympathetic, SIDS, sleep-disordered breathing, SUDEP, sympathetic

## Abstract

Central nervous system processing of autonomic function involves a network of regions throughout the brain which can be visualized and measured with neuroimaging techniques, notably functional magnetic resonance imaging (fMRI). The development of fMRI procedures has both confirmed and extended earlier findings from animal models, and human stroke and lesion studies. Assessments with fMRI can elucidate interactions between different central sites in regulating normal autonomic patterning, and demonstrate how disturbed systems can interact to produce aberrant regulation during autonomic challenges. Understanding autonomic dysfunction in various illnesses reveals mechanisms that potentially lead to interventions in the impairments. The objectives here are to: (1) describe the fMRI neuroimaging methodology for assessment of autonomic neural control, (2) outline the widespread, lateralized distribution of function in autonomic sites in the normal brain which includes structures from the neocortex through the medulla and cerebellum, (3) illustrate the importance of the time course of neural changes when coordinating responses, and how those patterns are impacted in conditions of sleep-disordered breathing, and (4) highlight opportunities for future research studies with emerging methodologies. Methodological considerations specific to autonomic testing include timing of challenges relative to the underlying fMRI signal, spatial resolution sufficient to identify autonomic brainstem nuclei, blood pressure, and blood oxygenation influences on the fMRI signal, and the sustained timing, often measured in minutes of challenge periods and recovery. Key findings include the lateralized nature of autonomic organization, which is reminiscent of asymmetric motor, sensory, and language pathways. Testing brain function during autonomic challenges demonstrate closely-integrated timing of responses in connected brain areas during autonomic challenges, and the involvement with brain regions mediating postural and motoric actions, including respiration, and cardiac output. The study of pathological processes associated with autonomic disruption shows susceptibilities of different brain structures to altered timing of neural function, notably in sleep disordered breathing, such as obstructive sleep apnea and congenital central hypoventilation syndrome. The cerebellum, in particular, serves coordination roles for vestibular stimuli and blood pressure changes, and shows both injury and substantially altered timing of responses to pressor challenges in sleep-disordered breathing conditions. The insights into central autonomic processing provided by neuroimaging have assisted understanding of such regulation, and may lead to new treatment options for conditions with disrupted autonomic function.

## Introduction

Regulation of the autonomic nervous system involves brain areas from the neocortex to the brainstem, as well as the cerebellum (Moruzzi, 1948; Miura and Reis, 1969). Those structures operate in a network; however, past autonomic studies have principally focused on subsets of forebrain and brainstem networks (Chase and Clemente, 1968; Benarroch, 1993). The objective of this review is to outline the widespread nature of brain structures involved in autonomic control as identified using neuroimaging procedures. A special emphasis will be placed on methodological aspects of neuroimaging, since the available techniques and their implementation influence what structures and types of function can be evaluated. Neuroimaging has confirmed the involvement of cerebellar and neocortical regions in autonomic regulation, which includes interactions with diencephalic areas including the hippocampus, basal ganglia, hypothalamus, as well as midbrain and brainstem regions. While lesion and stroke findings, as well as animal models have helped elucidate these roles, non-invasive, *in vivo* measurements of healthy brain activity using neuroimaging have consolidated our understanding of these structures' functions during normal and disrupted autonomic regulation. The most commonly used tool is functional magnetic resonance imaging (fMRI), where brain activity is measured every few seconds while participants undergo a challenge that elicits a change in autonomic state.

The earliest neuroimaging studies of autonomic function involved fMRI acquisition during blood pressure and sympathetic-activity induced by respiratory challenges. Although these early scanning protocols and analysis methodologies were exploratory in nature, the findings showed involvement of “widespread and lateralized” responses in multiple brain regions including limbic and cerebellar areas which were remarkably consistent with subsequent studies (Gozal et al., 1995b, 1996; Harper et al., 1998, 2000; King et al., 1999). Some of the early imaging studies of autonomic function involved position emission tomography (PET), an invasive technique that can be used to quantify blood flow and metabolism (Frackowiak et al., 1980). The early PET studies complemented the exploratory fMRI findings, demonstrating involvement of regions now known to assist central autonomic integration (Nowak et al., 1999; Critchley et al., 2000). Since a radioactive agent is required, and only single time-point measures of blood flow or metabolism are obtained, PET research studies are limited. The method is suited to metabolism issues, and concurrent measurements with instruments incompatible with high magnetic fields, such as muscle sympathetic nerve activity (MSNA) or continuous non-invasive blood pressure (Critchley et al., 2000; Krämer et al., 2014). Functional MRI offers more flexibility and avoids radiation exposure, and despite the disadvantage of being a relative measure (so that only changes in function can be detected), it has become the dominant modality in assessment of neural autonomic regulation, as reflected by the compilation of functional imaging studies of autonomic function in Table 1, and is the focus of this review.

**Table 1:** **Neuroimaging studies of autonomic function and their subject characteristics (in chronological order)**.

**Study**	**Challenges**	**Control/healthy group**	**Patient group**	**Technical**
Gozal et al., 1995b	Inspiratory loading	*N* = 11	n/a	fMRI
		Age 27–48 years		
Gozal et al., 1996	Expiratory loading	*N* = 11	n/a	fMRI
		1 female, 10 male		
		Age 28–46 years		
Harper et al., 1998	Expiratory loading; forehead cold pressor; hand cold pressor	*N* = 11	n/a	fMRI
		Age 22–55 years		
King et al., 1999	Hand grip, maximal inspiratory breath hold, Valsalva	*N* = 5 male	n/a	fMRI
		Age 19–46 years		
Nowak et al., 1999	Hand grip	*N* = 8	n/a	CBF with PET
		4 female, 4 male		
		Age 26 [23–30] years		
Critchley et al., 2000	Hand grip	*N* = 6 male	n/a	CBF with PET
		Age 31 ± 3 years		
Harper et al., 2000	Forehead cold pressor, hand cold pressor, expiratory loading	*N* = 11	n/a	fMRI
		3 female, 8 male		
		Age 22–37 years		
Henderson et al., 2002	Valsalva	*N* = 12	n/a	fMRI
		1 female, 11 male		
		Age 47 ± 10 [30–58] years		
Henderson et al., 2003	Valsalva	*N* = 15, male	OSA *N* = 8, male	fMRI
		Age 45 ± 12 [30–58] years	Age 45 ± 11 [31–63] years	
Harper et al., 2003	Forehead cold pressor	*N* = 16, male	OSA *N* = 10, male	fMRI
		Age 46 ± 12 [28–64] years	Age 47 ± 10 [30–60] years	
Macey et al., 2003b	Expiratory loading	*N* = 16, male	OSA *N* = 9, male	fMRI
		Age 46 ± 11 [29–63] years	Age 45 ± 12 [28–64] years	
Macey et al., 2004a	Expiratory loading	*N* = 14 children	CCHS *N* = 13 children	fMRI
		7 female, 7 male	6 female, 7 male	
		Age 10.9 ± 2.2 [8–15] years	Age 10.9 ± 2.3 [8–15] years	
Topolovec et al., 2004	Hand grip, maximal inspiration, Valsalva	*N* = 8	n/a	fMRI
		3 female, 5 male		
		Age 22–28 years		
Kimmerly et al., 2005	Lower body negative pressure	*N* = 8 male	n/a	fMRI
		Age 24 ± 2 years		
Macey et al., 2005a	Forehead cold pressor	*N* = 14 children	CCHS *N* = 14 children	fMRI
		7 female, 7 male	6 female, 7 male	
		Age 11.0 ± 2.2 [8–15] years	Age 10.9 ± 2.3 [8–15] years	
Macey et al., 2005b	Forehead cold pressor	*N* = 16 male	HF *N* = 6, male	fMRI
		Age 48 ± 11 years	Age 49 ± 12 years	
Macey et al., 2006	Inspiratory loading	*N* = 11 male	OSA *N* = 7 male	fMRI
		Age 47 ± 4 years	Age 46 ± 5 years	
Kimmerly et al., 2007	Lower body negative pressure	*N* = 16	n/a	fMRI
				
		8 female, 8 male		
		Age 22–24 ± 4 years		
Wong et al., 2007a	Hand grip	*N* = 17	n/a	fMRI
		9 female, 8 male		
		Age 24–25 ± 4 years		
Wong et al., 2007b	Hand grip	*N* = 17	n/a	fMRI
		9 female, 8 male		
		Age 25 ± 4 years		
Serber et al., 2007	Valsalva	*N* = 14 male	HF *N* = 5	fMRI
		Age 47 ± 11 years	2 female, 3 male	
			Age 50 ± 10 years	
Napadow et al., 2008	Hand grip	*N* = 7	n/a	fMRI with ECG gating
		3 female, 4 male		
		Age 21–33 years		
Goswami et al., 2011	Hand grip, muscle stimulation	*N* = 12	n/a	fMRI
		8 female, 4 male		
		Age 25 ± 3 years		
Goswami et al., 2012	Lower body negative pressure	*N* = 15	n/a	fMRI
		6 female, 9 male		
		Age 25 ± 3 [18–31] years		
Macey et al., 2012	Foot cold pressor, hand grip, Valsalva	*N* = 57	n/a	fMRI
		20 female, 37 male		
		Age 47.3 ± 8.8 years		
Ogren et al., 2012	Valsalva	*N* = 33	HF *N* = 16	fMRI
		10 female, 23 male	5 female, 11 male	
		Age 52.3 ± 7.7 years	Age 54.4 ± 8.1 years	
Kimmerly et al., 2013	End-expiratory breath hold, Mueller maneuver	*N* = 16	n/a	fMRI
		9 female, 7 male		
		Age 35 ± 14 [20–65] years		
Norton et al., 2013	Hand grip	*N* = 29 (“responders”)	*N* = 15 (“non-responders”)	fMRI
		11 female, 20 male	7 female, 8 male	
		Age 48 ± 19 years	Age 56 ± 19 years	
Richardson et al., 2013	Cold pressor	*N* = 24 adolescents	n/a	fMRI
		11 female, 13 male		
		Age 15.5 ± 2.0 [11–18] years		
Macey et al., 2014	Foot cold pressor, hand grip, Valsalva	*N* = 57	OSA *N* = 37	Global BOLD
		20 female, 37 male	6 female, 31 male	
		Age 47.3 ± 8.8 years	Age 53.9 ± 8.4 years	
Bohr et al., 2015	Valsalva	*N* = 45 elderly	n/a	T2^*^ fMRI
		24 female, 21 male		
		Age median 80 [75–89] years		
Norton et al., 2015	Hand grip	*N* = 23	*N* = 17 coronary artery disease	fMRI
		8 female, 15 male		
			4 female, 13 male	
		Age 63 ± 11 years	Age 59 ± 9 years	
Wu et al., 2015	End-expiratory and end-inspiratory breath hold, Valsalva	*N* = 9	n/a	fMRI
		4 female, 5 male		
		Age 29 ± 8 years		

*Relevant challenges are noted. Ages are mean ± sd [range] where available. “fMRI” refers to BOLD fMRI*.

We will review methodological aspects of testing central autonomic function, and present research findings in healthy and pathological conditions, including obstructive sleep apnea (OSA), congenital central hypoventilation syndrome (CCHS), and heart failure (HF), all of which are linked to impaired autonomic function. Some autonomic circuitry is highlighted, with other information available elsewhere (for example, Shoemaker and Goswami, 2015). Although mental and emotional stressors are closely linked with autonomic function, the scope of this paper is restricted to stimuli that are principally physical, rather than psychological. Pain is also not within the scope of this review, since that phenomenon encompasses more aspects than just autonomic regulation. This manuscript focusses on active autonomic testing, as opposed to resting state analyses of brain activity for which the reader is directed elsewhere (for example, Henderson et al., 2012; James et al., 2013; Macefield et al., 2013). Although we focus on fMRI methodology in this review, emerging neuroimaging measures that offer potential for addressing future research questions are also briefly discussed.

## Functional MRI methodology

### Types of autonomic function that can be assessed with fMRI

Several standard autonomic tests can be adapted to the MRI environment. Autonomic challenges usually consist of physical stimuli or actions that elicit changes in sympathetic and parasympathetic activity, often reflected as blood pressure and heart rate alterations. While certain tests are impractical inside an MRI environment (such as orthostatic changes elicited either by a tilt table or by changing posture from sitting to standing: Faraji et al., 2011; Saal et al., 2015), the majority of procedures involving a static body position with minimal electrical equipment are feasible (Adkisson and Benditt, 2015). The Valsalva maneuver can be performed in the supine position, with plastic tubing connected to a pressure sensor outside the scanner room and some form of signal/cue to indicate to the participant when to blow into the tube, and when sufficient pressure is reached. Other respiratory-related tasks that can use a similar tubing setup are expiratory and inspiratory loading. The breath hold requires only a means to signal to the participant when they should begin and end their breath hold period, which can be as simple as the scanner operator giving instructions through the audio link. Both inspiratory capacity apnea and end-expiratory breath-hold challenges elicit sympathetic activation (Macefield and Wallin, 1995; Zubin Maslov et al., 2014). A hand grip, which also involves sympathetic activity increases (Saito et al., 1986), requires a non-magnetic device such as a squeeze bulb or custom MRI-compatible manometer, and some method of indicating to the participant when and how hard to grip. Baroreceptor unloading tasks include lower body negative pressure (Sundlöf and Wallin, 1978; Victor and Leimbach, 1987; Kimmerly et al., 2005; Goswami et al., 2012), which requires a specialized suit, and the simpler-to-implement Mueller maneuver (Somers et al., 1993; Kimmerly et al., 2013). The cold pressor is a passive test which has the advantage of being a consistent stimulus across subjects (Victor et al., 1987), although the pain component is a confound to the blood pressure manipulation (Peckerman et al., 1991, 1994). A forehead cold pressor, used to stimulate the trigeminal nerves and dive reflex, requires the use of near-frozen, non-polarized heavy water (deuterium) to avoid scanner artifact (Harper et al., 2003; Macey et al., 2005a). Foot or hand cold pressor tests require a plastic container with cold water (and towels to avoid water spilling; Valladares et al., 2006). Head movement at the beginning and end of a cold pressor test is common, and analyses must account for these effects. Functional MRI is less suitable for identifying changes in state that last several minutes, such as the quantitative sudomotor axon reflex test. However, electrical stimulation of muscle has been performed, with appropriate safety precautions (Goswami et al., 2011).

**Figure 1:**
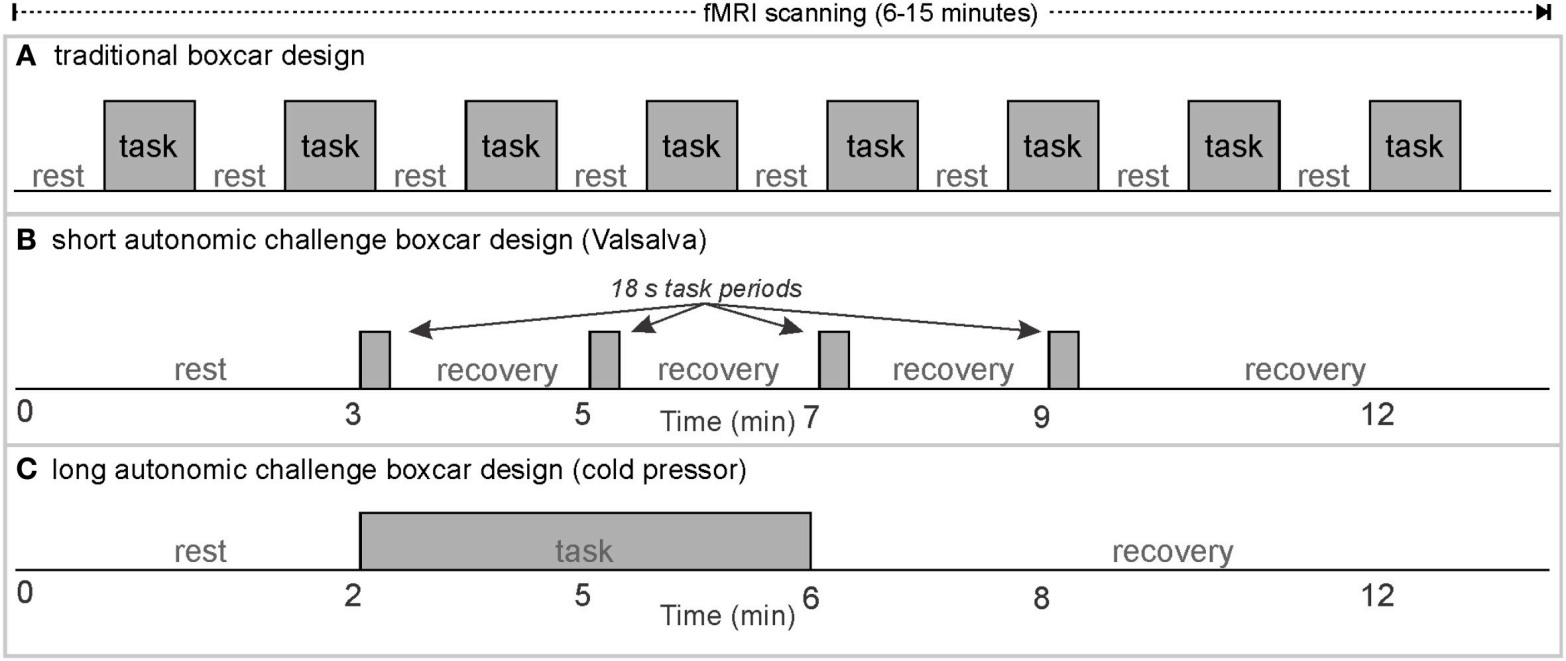
**Classical boxcar design for fMRI scanning; examples of rest/task timing for (A) classical visual or motor stimulus paradigms, (B) short autonomic challenges, and (C) long task/recovery duration autonomic challenges**.

The Valsalva maneuver has been the focus of particular attention as a simple challenge that elicits a strong autonomic reaction (Hamilton et al., 1936). The task can be performed in an MRI scanner (King et al., 1999; Henderson et al., 2002, 2003; Serber et al., 2007; Woo et al., 2007; Ogren et al., 2010, 2012; Macey et al., 2012), and can be repeated multiple times within the timeframe of a typical fMRI protocol (Figure 1). This task has been proposed as a potential method for assessing the baseline vascular status in fMRI studies to control for cerebral blood volume and oxygenation differences across subjects (Wu et al., 2015), and investigating brain areas susceptible to hypoxia during autonomic challenges (Bohr et al., 2015). Our group has used this challenge to demonstrate neural, cerebrovascular, and peripheral alterations in healthy and patient groups (Henderson et al., 2002, 2003; Serber et al., 2007; Macey et al., 2013, 2014). Additionally, the robust activation elicited by the Valsalva has allowed distinct sub-regions of the insula to be functionally separated (Macey et al., 2012).

Practicing autonomic tasks prior to scanning is important for reliable assessment of autonomic neural responses. Practice outside the MRI and again inside the bore of the scanner minimizes the novelty effect, which could otherwise introduce a confound (Kelly and Garavan, 2005). Practicing outside the MRI also allows participants to learn the task more effectively, since the confined space within the scanner bore is often stressful, and the required ear protection makes understanding audio instructions more difficult. We typically practice outside the scanner, then on the scanner bed outside of the bore, and finally when the bed is moved inside the bore. We also avoid performing an fMRI sequence immediately after practice, since blood pressure effects of some challenges can last for minutes, so we intersperse the practice and functional sequences with structural scans.

### Task-related cerebral blood volume and oxygenation confounds

Most autonomic challenges lead to blood pressure or oxygenation changes that influence the fMRI signal. These effects are effectively independent of neural activation-related changes (Kim et al., 1999), and thus, are usually excluded as confounds from fMRI analyses (Macey et al., 2004b). Cerebral blood volume is related to cerebral blood flow, closely linked with tissue oxygen demand, and blood vessel volume, influenced by vasodilatory or vasoconstrictive influences from blood gas levels and blood pressure (Kety and Schmidt, 1948; Kety et al., 1948; Grubb et al., 1974; Ito et al., 2005; Tasker, 2013). While these influences are considered global in nature, that is, occurring similarly across the whole brain, white matter has substantially less CBF but more CBV, so changes in these variables would likely differ by tissue type (Schreiber et al., 1998). Analytic approaches that assume a constant influence across the intracranial space (such as intensity normalization, which involves setting each fMRI volume to have the same average intensity) lead to inaccuracies (Macey et al., 2004b). For example, since intensity normalization assumes the contribution of global confounds to the fMRI signal are considered equal in gray and white matter, the greater contribution of white vs. gray matter will lead to globally-related signal increases in gray matter and decreases in white matter. Neural activation occurring independently of the task-induced global effects can be viewed by removing all confounds with a method such as “LMGS” (Macey et al., 2004b). Alternatively, differences in neural activation between different brain regions can be measured as differences in fMRI signal between those regions (Macey et al., 2012), since the global effects should be constant across gray matter structures.

### Task-related neural activation confounds

The nature of a particular active or passive challenge to the autonomic nervous system leads to distinct neural responses. Active challenges require voluntary participation, typically recruiting unique sensory, cognitive, decision, and motor systems, as in squeezing for a hand grip task, or forced expiration for the Valsalva. Passive challenges require no participant action, and include such manipulations as lower body negative pressure. The cold pressor can be active (participant moves foot or hand into cold water) or passive (experimenter moves limb into cold water, or applies forehead cold pack). The active Valsalva maneuver requires participants to wait for a cue (visual processing, attention, and decision making), recruit respiratory musculature with forceful expiration (motor) while observing a level indicator and maintaining an appropriate pressure (visual processing, cognitive processing, decision making), and wait for a cue to end the task. Both active and passive challenges involve sensory input from the challenge, such as the sensation of cold and pain in the foot for a foot cold pressor. The pain component in a cold pressor challenge is an important confound—this task is often used as a pain, rather than pressor challenge in other contexts (for example, La Cesa et al., 2014). Using a temperature above freezing can help reduce the pain component while still providing a blood pressure stimulus; we use 4°C (Harper et al., 2003; Macey et al., 2005a). For any protocol, interpretation of fMRI activations should include consideration of non-autonomic, task-related influences.

### Scanning and protocol methodology

Functional MRI has been used to indirectly measure neural activity for over two decades (Ogawa et al., 1990). The technique involves measuring changes in blood volume and oxygenation in a timeframe of seconds. The measure, termed the blood-oxygen level dependent (BOLD) signal, has been validated as a marker of changes in neural activity (Logothetis et al., 2001). The neural activation regional BOLD response is robust even under conditions that strongly altered cerebral blood flow and volume. Although publications report significant alterations in the BOLD activation with varying baseline state, a detailed assessment of findings in one of the well-regarded comprehensive assessments shows that the general pattern is robust even to high levels of 5% CO_2_ hypercapnia (for example, Figure 1 in Cohen et al., 2002), with only moderate variations in timing and amplitude. Considering 5% CO_2_ as one of the most impactful stimuli on global blood volume and oxygenation, which leads to a 5% increase in the global BOLD signal (Cohen et al., 2002), the autonomic challenges typical used in fMRI studies result in substantially lower global influences, with for example, the Valsalva leading to a maximum global BOLD change of ~1%, and hand grip and cold pressure to less than 0.5% (Macey et al., 2014). Thus, the impact of a particular challenge on BOLD timing and magnitude is likely to be modest. However, the analytic challenge of distinguishing global from neural effects remains (see confounds above). Technical characteristics of fMRI protocols constrain the types of experimental questions that can be evaluated, and are briefly reviewed. An extensive literature is available elsewhere (for example, Amaro and Barker, 2006); thus, only aspects relevant to autonomic testing are addressed here.

#### The fMRI measure: blood-oxygen level dependent (BOLD) signal

The BOLD signal is not a quantitative measure, and does not measure absolute levels of blood oxygenation, but rather changes in neural activity (Ogawa et al., 1993). Experiments using fMRI to detect neural control of a particular behavior or internal state must, therefore, be designed to elicit changes in neural activity, which would then be reflected as alterations in the BOLD signal. Resting state fMRI is an alternative methodology used to assess spontaneous, resting neural activity based on spontaneous fluctuations in the BOLD signal (Vanderwal et al., 2013). Typical fMRI paradigms are of a “boxcar” design with stimuli or challenges presented multiple times with intervening rest periods (Figure 1A; Friston et al., 1995). Such paradigms work well for tasks with rapid, “on/off” type responses, such as visual or auditory stimuli, or rapid cognitive processing tasks (Engström et al., 2004). A standard paradigm begins with a rest period, and is followed by repetitions of the same task (or presentation of stimuli) separated by rest periods, usually ending with a final rest period. The “boxcar” shape is typically the expected pattern of activation of the areas of interest. For example, if the task consists of a dynamic visual stimulus, and the rest period of observing a static “+” (termed a fixation cross), neural activation in regions of the visual cortex would be predicted to follow the boxcar shape, that is, higher during task than rest, with rapid transitions at task onset and termination. The BOLD signal measures increases and decreases in neural activity-related blood volume and oxygenation levels, with results typically represented as percent change from the initial rest period (baseline). Correlational designs, which include resting state fMRI, require variation in the independent variable and fMRI signal; if no changes in neural activity occur in a particular region, fMRI cannot detect whether the tonic activity level was high or low. Regardless of the neural function being assessed, at least two conditions (one of which could be rest), are required for fMRI designs.

#### Brain activity at rest

Since the concept of activation relative to rest is common to many autonomic fMRI protocols, the questions of what is a resting state is briefly addressed. In boxcar analyses, the rest period is assumed to be a constant level of activity. However, the brain at rest is not in a constant state, and exhibits fluctuations, on occasion of a periodic nature. Such fluctuations vary by region, and resting state fMRI (rs-fMRI) is a technique that identifies networks of brain areas that show correlated activity fluctuations (Lee et al., 2013); these networks are defined as being functionally connected (Cordes et al., 2001). One consequence of the relative nature of BOLD fMRI is that regions connected functionally on a neuronal level, but not showing fluctuations, will not be identified by rs-fMRI. Nevertheless, assessment of functional connectivity with rs-fMRI has highlighted networks that closely overlap autonomic regions, such as the “default mode network” (the set of regions showing the strongest correlation, as identified by the principal component of an independent components analysis; Greicius et al., 2003), and especially the “salience network” (Seeley et al., 2007). The networks identified by rs-fMRI show changes in conditions of altered autonomic function including OSA (Santarnecchi et al., 2013; Zhang et al., 2013, 2015; Park et al., 2014, 2015), and are sensitive to changes in brain function occurring with treatment in longitudinal studies (for example, Fu et al., 2015). Considering fMRI protocols for autonomic testing (or any task-related protocol), the spontaneous variability in neural activity means that baseline periods should be sufficiently long to minimize the error due to the intrinsic variation, and activation should be large enough to dominate over resting state patterns.

#### Brain activity in response to a challenge

**Figure 2:**
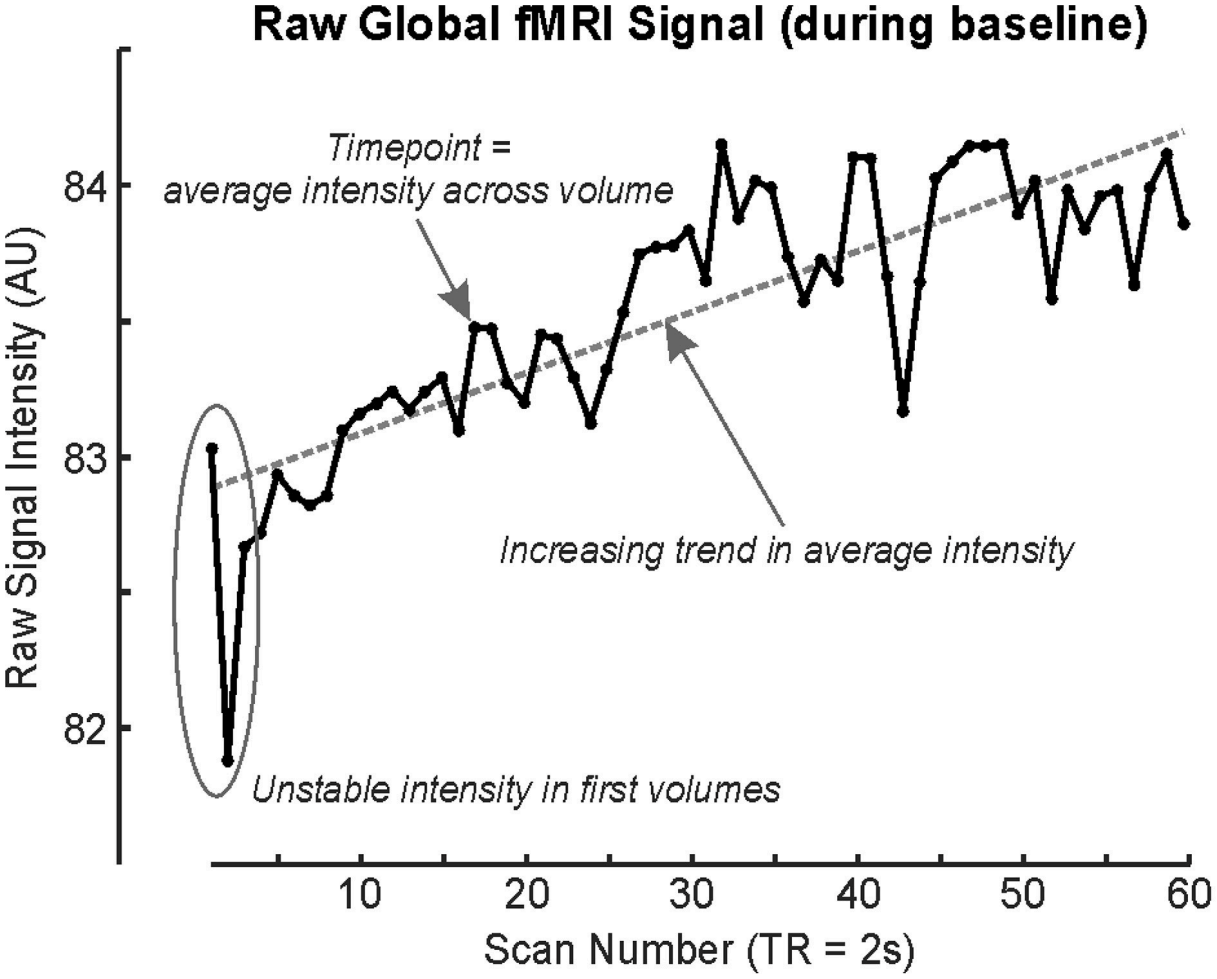
**fMRI time series' global signal illustrating drift during a baseline (rest) period, with each timepoint representing the average signal intensity across the whole brain volume (scan)**. First fMRI scans are usually rejected due to time taken for the signal to stabilize (although modern sequences do this automatically; Data from single subject baseline of cold pressor in Macey et al., 2012).

Neural patterning regulating autonomic output does not follow a simple on/off pattern, just as somatomotor or sensory response patterns are complex. The Valsalva maneuver is one autonomic challenge with multiple response phases (Goldberg et al., 1952; Porth et al., 1984), influences, by varying degrees, both sympathetic and parasympathetic outflow. Despite consisting of a relatively simple somatomotor task, namely exhaling against a resistance to a predetermined pressure (30–40 mmHg) for a defined period (15–20 s), the cardiovascular response is separated into four distinct patterns occurring over periods of time, or phases, not including preliminary inhalation. Phase 1 and 3 are short (<5 s), whereas phase 4 lasts up to 4 min. Phase 4 is a return to baseline post-task, and for practical reasons, is usually assumed to be complete after 1–2 min; thus, a repeat of the Valsalva can be performed within the same fMRI series (A “series” is a single, continuous acquisition of fMRI scans). As the fMRI signal is in arbitrary units (Figure 2), repeated tasks must be performed within the same acquisition series if they are to be analyzed together.

#### Timing of autonomic challenges

Since autonomic challenges typically involve changes in blood pressure and heart rate that persist over tens of seconds to minutes, considering the classical design in Figure 1A, each block of an autonomic task, and especially “rest” would ideally have a duration in the order of 1–5 min, and thus, the number of repeated tasks needs to be limited to keep the paradigm within the typical 5–15 min fMRI protocol. Examples of autonomic paradigms include three or four Valsalva maneuvers separated by (ideally) at least 1 min (Figure 1B), static hand grip tasks of up to 2–4 min, and cold pressor challenges with a single 1–2 min challenge period (Figure 1C).

Both technical and logistical issues limit long protocols (15 min and longer). The BOLD signal is not stable over a timeframe of minutes due to heating of the MRI scanner coils (Figure 2); fMRI scanning involves stimulating protons with radiofrequency pulses as well as manipulating gradients, and these actions involve energy transmission. Since the order of magnitude of BOLD signal changes associated with neural activation is in the order of 1% (Logothetis et al., 2001), small effects on the underlying signal can affect the outcome. Some longer paradigms have been used to assess sleep state-related neural activity, but these require specialized preprocessing techniques, as in the 40 min protocol by Fukunaga et al. (2006). Practical limitations include the difficulty in having a research subject remain attentive to the task, or comfortably lying immobile. Cost may also be an issue, since MRI scanning costs are typically time-dependent.

#### Temporal resolution

The time resolution of most current fMRI scanning is 2 s/whole brain volume; the time to collect one volume is termed “repetition time,” or TR. The TR is limited by hardware and more recently, software factors. The strength of the main magnetic field in the MRI, the speed of turning gradient coils on and off, and the maximum magnetic field gradients create hardware constraints. Sensitivity of the coil is influenced by the number of channels, and the use of digital signal processing and transmission in recent scanners. Approaches to parallel acquisition using multiple coils and multiple energizing frequencies require a combination of hardware and software to allow for low TR's (Sodickson et al., 2005).

Earlier technology (late 1990's and early 2000's) allowed down to a 6 s TR, with often only partial brain coverage. The period from the mid-2000's to the present has seen whole-brain coverage with TR's of 2 s at voxel sizes of ~2.5 × 2.5 × 3.5 mm, and many studies have been performed at this resolution. State-of-the-art multiband techniques available on modern clinical MRI scanners from 2015 allow for 0.7 s TR's, and a seven-fold increase in spatial resolution (Tomasi et al., 2015). Since the hemodynamic response is relatively, slow varying, regardless of the speed of change of underlying neural activity, sub-second TR's are reaching the limit of what is needed to fully sample signals representing activation, at least as understood at present. However, since autonomic regulation involves numerous brain areas operating in a network, identifying the *timing* of responses of one region relative to another would give phase and direction information. Current fMRI technology easily allows identification of whether a particular region shows activation to a resolution of 2 s, but knowing when the activation began to a resolution of at least 100's of milliseconds would be required to see which brain regions initiate higher-level network activity, and the progression of responses over time. However, the timing between nuclei in basic circuits like the baroreflex network is well under 10 ms (Sabatini and Regehr, 1999), which is out of reach of both scanning resolution and hemodynamic response specificity. Improved resolution is possible on a slice-by-slice basis; over a 2 s TR, typically more than 40 slices will be collected, which requires 50 ms/slice (Konn et al., 2004). The limitation of such an approach is that only brain regions visible within one slice can be assessed at a resolution of 50 ms, but nevertheless, this targeted approach could allow testing of hypotheses generated from whole-brain findings.

#### Spatial resolution

Spatial resolution of fMRI is suited to identifying neural activity in cortical areas and larger structures. Some brainstem nuclei cannot be easily spatially distinguished, but robust activation in regions several millimeters across can be detected. Focusing the fMRI acquisition on specific regions can enhance both spatial and temporal resolution as discussed above. For example, brainstem-specific scans allow higher resolution (<2 mm; Macefield and Henderson, 2010). In some scanners, brainstem protocols show fewer artifacts if the standard axial acquisition is changed to coronal. Current state-of-the-art approaches allow 1.5 mm^3^ resolution at a fast TR (Tomasi et al., 2015). A spatial limitation additional to the small size of brainstem nuclei is that the hemodynamic response is a diffuse phenomenon, limiting spatial differentiation of the BOLD signal. Prior to analysis, fMRI scans are typically smoothed with a Gaussian filter with an 8 mm kernel, down to 4 mm for brainstem scans, and these kernel sizes give some indication of the spatial resolution of the hemodynamic response.

#### Methodology summary

In summary, protocols specific to autonomic testing require consideration of potential confounds and limitations of fMRI methodology, with total time of the entire sequence and the spatial and temporal resolution essential issues. Even within the current technical constraints, studies to date have demonstrated the involvement of multiple regions during autonomic actions. The improved spatio-temporal specifications available with recent scanning developments have made possible assessment of sequencing of activated patterns between structures, opening many opportunities to determine organization of autonomic function.

### Analytic issues: expected pattern of response vs. expected region responding

**Figure 3:**
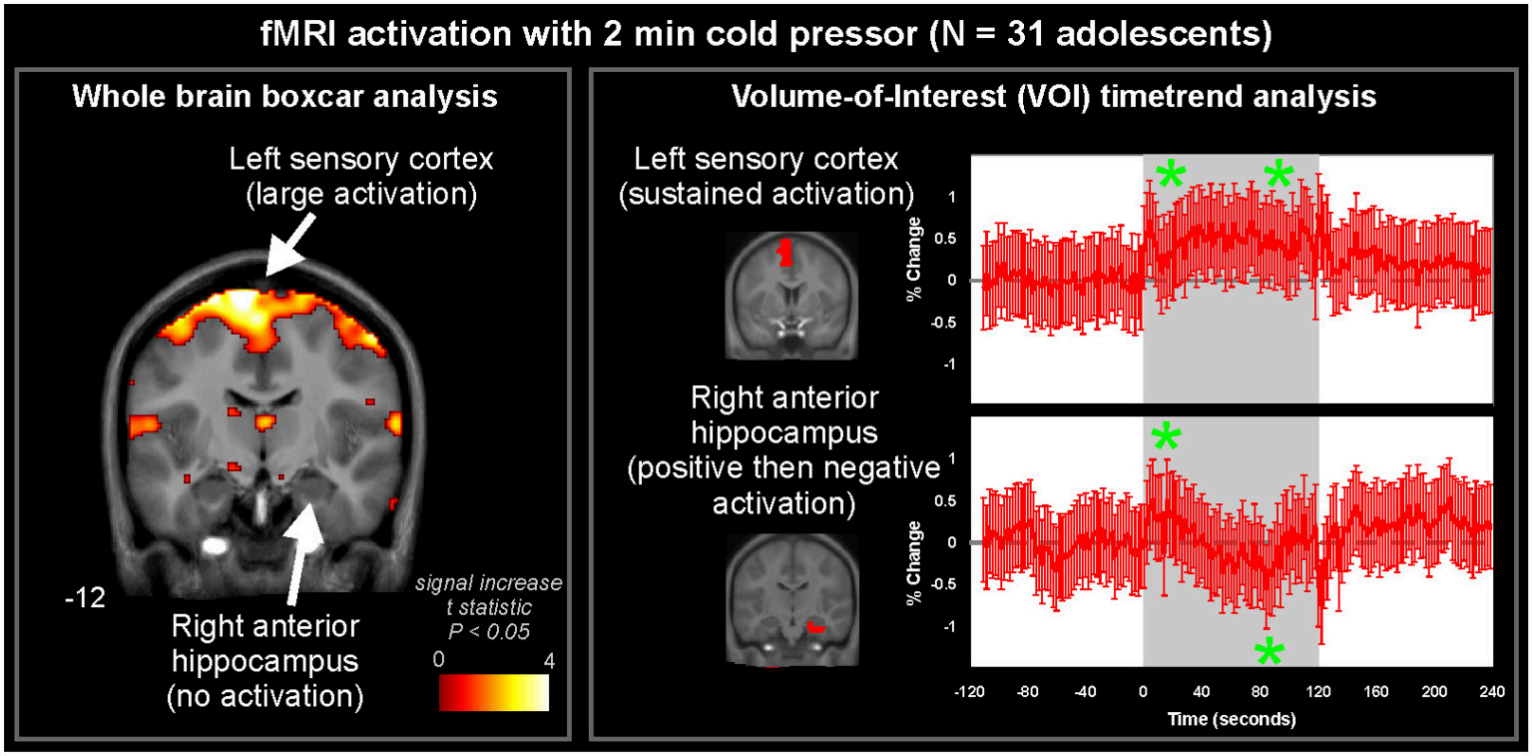
**Whole-brain and volume-of-interest analysis of fMRI signal during 2 min cold pressor in 31 healthy adolescents (age 15.0 ± 2.5, 14 female/17 male). Left:** A whole-brain analysis of activations fitting a pre-determined pattern (boxcar, as in Figure 1C) highlights the sensory cortex and adjacent areas (pseudo-colored areas of significance overlaid onto anatomical background), but not right hippocampus. **Right:** Timetrend analysis of VOI based on pre-determined regions derived from the Automated Anatomical Labeling atlas (Tzourio-Mazoyer et al., 2002), highlighting significant activations (indicated by ^*^) in both the sensory cortex and right hippocampal area (repeated measures ANOVA, *P* < 0.05) (Data from Valladares et al., 2006).

Two general approaches to analyzing fMRI autonomic response are assessing the whole brain with a pre-determined model, or assessing the time-course in a predetermined region or volume-of-interest (VOI). A third alternative is a model-free approach, which is discussed later in the “Other Analytic Methodologies” section; this approach has been little used in autonomic neuroimaging. The time-varying nature of autonomic regulation means traditional boxcar models of neural activation are not always well-suited to identifying response patterns. An illustration of these two approaches is in Figure 3, which presents results from a 2 min right foot cold pressor in adolescents (Valladares et al., 2006). The boxcar analysis using SPM software (Friston et al., 1995) highlights the contralateral foot sensory region and various adjacent areas as activating during the challenge. However, the anterior right hippocampus, while not highlighted by the SPM analysis, shows a pattern of response that is elucidated with a timetrend from the fMRI data averaged over the VOI; this hippocampal timecourse consists of an initial increase followed after 1 min by a transient decrease in activation, with a return toward baseline by the end of the 2 min challenge. The timecourse of a VOI of the precentral gyrus shows a timetrend increased from baseline throughout the challenge period, as in the boxcar share in Figure 1C, confirming the “cluster” SPM finding. These and related findings are discussed from a scientific perspective below, but this example (Figure 3) highlights the analytic issue of being limited to a pre-defined pattern of response with a standard SPM approach, or a pre-defined region with the timetrend approach; both approaches may be warranted for a challenge.

## Findings of normal function

We performed a series of studies in healthy people using the Valsalva maneuver, hand grip, and cold pressor challenges. Functional MRI during these tests has highlighted the multiple brain regions involved in regulating autonomic action. A major contribution of neuroimaging has been to demonstrate the widespread nature of central autonomic regulation, extending from the neocortex to the brainstem and cerebellum, often in a lateralized manner, and following a temporal sequence of recruitment in different brain areas. While the methodological issues, such as blood pressure and oxygenation influences may confound individual findings, as discussed above, the consistency of results across varying challenge types, scanning protocols, and subject populations provides reassurance of the interpretation of the findings as reflecting neural activation.

### Brainstem responses

**Figure 4:**
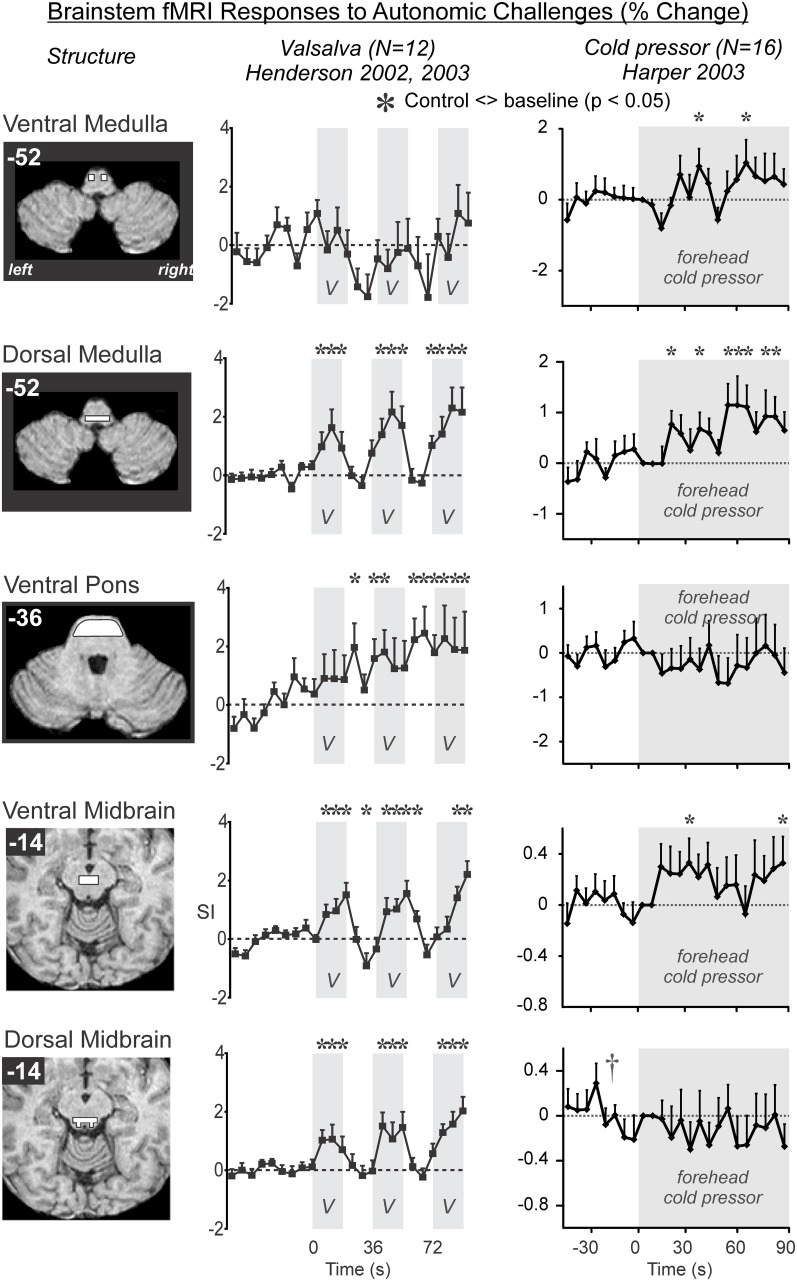
**Brainstem involvement during autonomic challenges detected by fMRI**. A “^*^” symbol indicates a timepoint of significant signal increase relative to baseline, by repeated measures ANOVA at *P* < 0.05 (Data from Henderson et al., 2002; Harper et al., 2003; Henderson et al., 2003).

**Figure 5:**
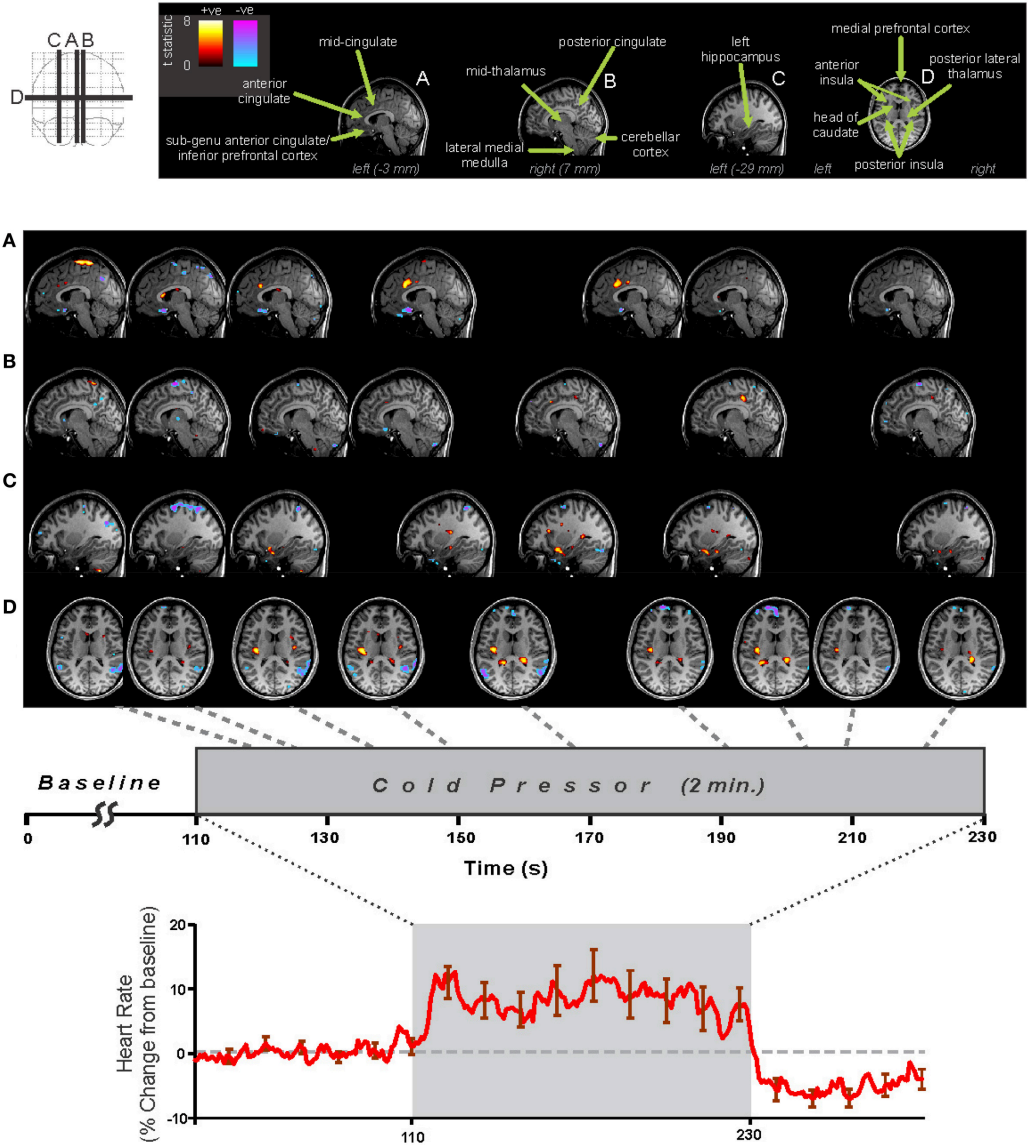
**Foot cold pressor fMRI and heart rate responses in 29 healthy adolescents (16 male, age mean ± sd [range] = 15.3 ± 2.4 [10.1–19.0] years, 4 left handed)**. Analysis using SPM software with timepoint-by-timepoint modeling of significant responses (each point *p* < 0.05 *t*-test, all regions corrected at whole-model *F*-test level for multiple comparisons at *p* < 0.05 family-wise error). Heart rate calculated from pulse-oximetry signal. Regions of signal increases (warm colors) and decreases (cold colors) are overlaid on three sagittal **(A–C)** and one axial **(D)** slices at selected time-points during challenge (Data from Valladares et al., 2006).

**Figure 6:**
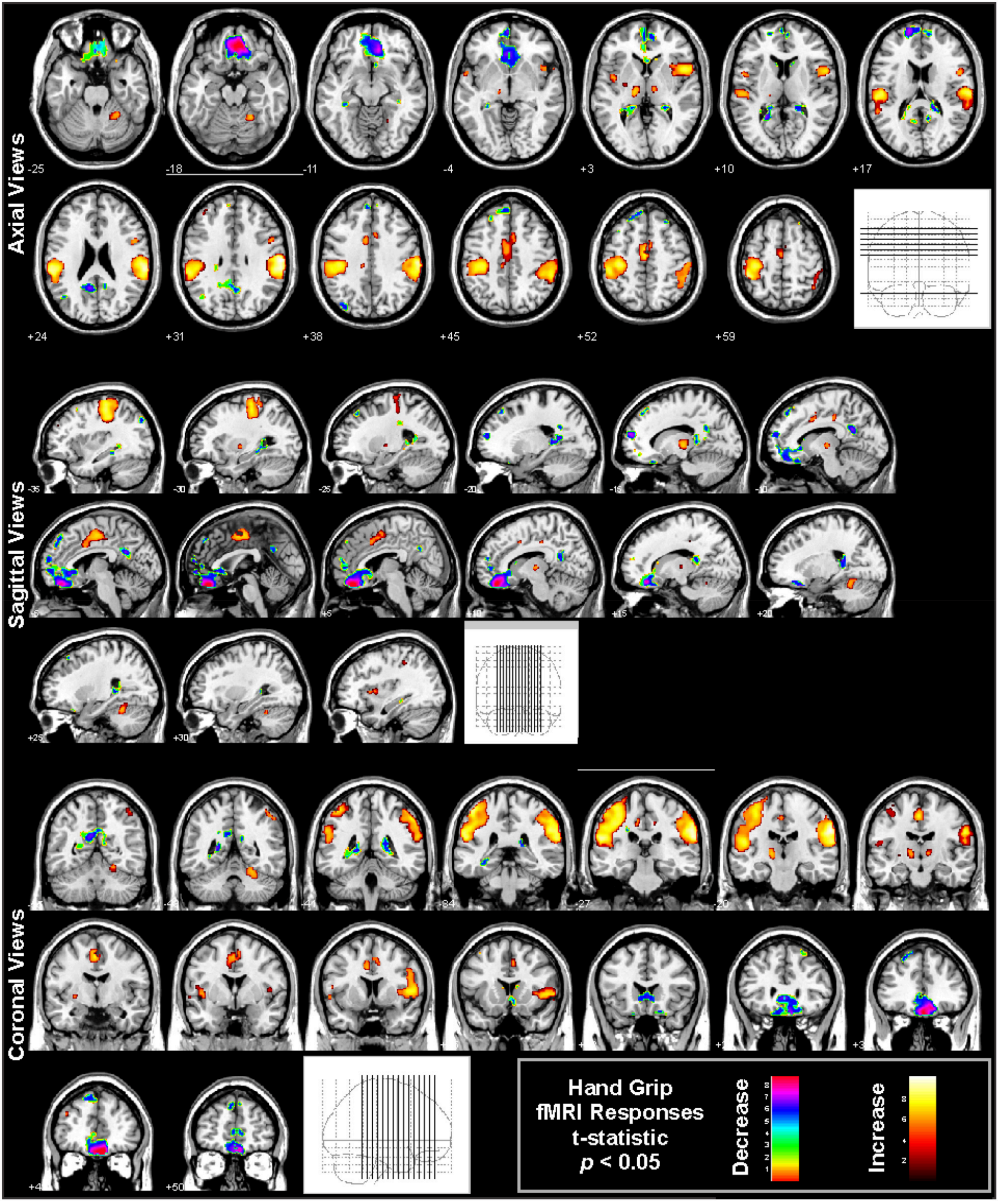
**Hand grip fMRI responses in 65 healthy controls (41 male, age mean ± sd [range] = 47.5 ± 8.8 [30.9–65.8] years, 11 left handed)**. The protocol consists of four 16 s challenges of 80% subjective maximal grip, 1 min apart. Analysis using SPM software with boxcar modeling of significant responses (*p* < 0.05 *t*-test, all regions corrected at whole-model *F*-test level for multiple comparisons at *p* < 0.05 family-wise error). Regions of signal increases (warm colors) and decreases (cold colors) are overlaid onto multiple views, illustrated the varied regions of response (Data from Harper et al., 2008).

The pathway for sympathetic outflow in the ventral medulla is well-described (Loewy, 1982; Strack et al., 1989), and the temporal patterns for such medullary activation on fMRI to foot cold pressor and Valsalva maneuver are readily apparent in healthy adolescents and adults (Figure 4; Henderson et al., 2002, 2003; Topolovec et al., 2004; Ogren et al., 2010; Richardson et al., 2013). The extent of activation may vary with the particular cold challenge; a forehead cold pressor increases activation in the dorsal medulla, with more modest changes in the ventral medulla and midbrain (Figure 4; Harper et al., 2003), while in children, the forehead cold pressor is associated with detected increased activity in the midbrain and pons, but not medulla (Macey et al., 2005a), suggesting the pattern of response in the medulla may vary (since the analytic method used was designed to detect sustained, boxcar-like activation only; see Figure 4). In children and adults, similar responses are elicited to forced expiratory loading, a sustained form of the Valsalva maneuver (Macey et al., 2003b, 2004a). A 2 min static hand grip similarly shows activity in multiple brainstem regions consistent with nuclei involved in blood pressure regulation (Sander et al., 2010). Visualization of significant responses to cold pressor (Figure 5) and hand grip (Figure 6) using whole-brain boxcar analyses show no brainstem activation, but this is likely due to the analytic method used (see Figure 3 and above), since VOI analyses show medullary activation to the cold pressor in healthy adolescents (Richardson et al., 2013).

### Central autonomic network and limbic regions

A substantial body of animal and human evidence from recording, lesion, stroke, and physiological studies demonstrate that cortical brain regions and other rostral brain areas participate in autonomic regulation (Chapman et al., 1949; Kaada et al., 1949; Oppenheimer and Cechetto, 1990; Oppenheimer et al., 1992, 1996; Benarroch, 1993; Al-Otaibi et al., 2010). Neuroimaging has confirmed these original findings, and extended the regions we now know to be involved in autonomic regulation.

**Figure 7:**
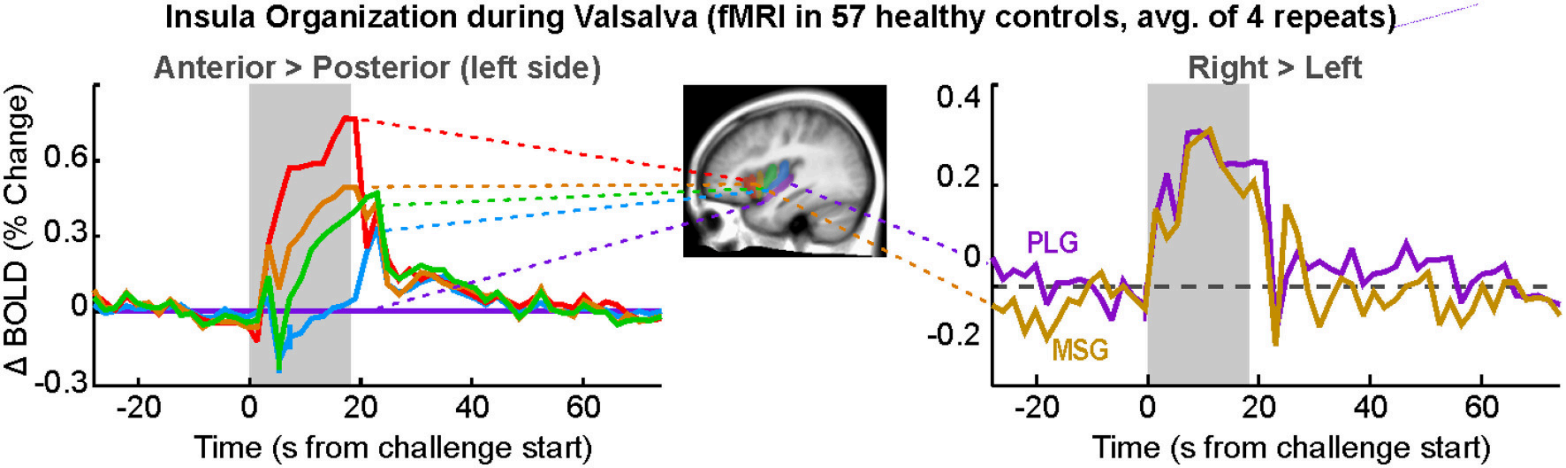
**Insular gyri organization during responses to Valsalva maneuver (simplification of findings in Macey et al., 2012). Left:** gyral activation relative to posterior-most gyrus (posterior long gyrus; PLG) on the left side, with anterior-most gryi showing great activation over posterior areas. **Right:** greater activation in right over left insular cortex in anterior and posterior gyri (mid short gyrus [MSG], PLG). Differences are significant (*P* < 0.05; Data from Macey et al., 2012).

Among the cortical areas, the insula participates in blood pressure challenges in a significant fashion. Forehead cold pressor, lower body negative pressure, the Valsalva, and the related forced expiratory loading lead to insular activation in adults and children (Henderson et al., 2002, 2003; Harper et al., 2003; Macey et al., 2003b, 2005a, 2012; Kimmerly et al., 2005, 2007; Shoemaker et al., 2015). Hand grip and maximal inspiratory loading similarly recruit the anterior and posterior insula (King et al., 1999; Macefield et al., 2006; Sander et al., 2010; Goswami et al., 2011, 2012). However, end-expiratory breath-hold and the Mueller maneuver result in some areas exhibiting declining signals, suggesting deactivation in subregions of the insula during those challenges (Kimmerly et al., 2013), i.e., a potential disfacilitation from the insula over other areas. Current fMRI protocols allow for separation of the insula into subregions based on major gyri, and the anterior-most gyri show greater responses during sympathetic stages of challenges, specifically the foot cold pressor, hand grip, and Valsalva (Figure 7; Macey et al., 2012). The insula has inhibitory projections to the hypothalamus, which likely form a major regulation pathway (Allen et al., 1991). Furthermore, the functional organization of the insula is asymmetrical, with the right side being preferentially active during sympathetic increases and the left side during parasympathetic action (Oppenheimer and Cechetto, 1990; Oppenheimer et al., 1992, 1996; Oppenheimer and Hachinski, 1992). This right-sided sympathetic preference was originally proposed based on animal studies and human stroke findings (see previous citations), but neuroimaging confirms that during the sympathetic phase of the Valsalva (phase II), the right insula is more active than the left (Figure 7; Macey et al., 2012).

Early stimulation studies showed that the cingulate is involved in blood pressure regulation (Pool and Ransohoff, 1949), and neuroimaging has demonstrated that specific subregions are recruited to cold pressor challenges. The mid-cingulate, in particular, responds to blood pressure changes (Macefield and Henderson, 2015), sometimes decreasing activation during sympathetic phases of the challenges, which could be associated with declining vagal tone. Detailed assessments of cold pressor and hand grip responses also show involvement of the anterior and posterior sections of the cingulate (Figures 5, 6).

Other regions involved in autonomic regulation are the ventromedial prefrontal cortex (VMPFC), basal ganglia, and hypothalamus, along with the amygdala and hippocampus, as shown in some of the earliest fMRI studies (Harper et al., 1998, 2000). The hypothalamus plays a major role in regulating autonomic outflow (Palkovits, 1999), with substantial projections from other limbic structures and efferent projections to the brainstem (Smith and Clarke, 1964). The hypothalamus shows fMRI signal responses under some conditions (Harper et al., 1998, 2000; Sprenger et al., 2004), but the structure is small, and differentiating local responses of the multiple subnuclei of the hypothalamus is difficult. However, fMRI has shown the VMPFC as well as amygdala and hippocampus play significant roles in the sequencing of responses to blood pressure and other challenges (Figures 5, 6; Wong et al., 2007b; Goswami et al., 2011); stimulating the hippocampus has long been known to lead to large changes in blood pressure (Ruit and Neafsey, 1988). The VMPFC activity during autonomic actions appears closely related to hippocampal function (Norton et al., 2013), and the hippocampus often responds to blood pressure and other challenges involving sympathetic activation (Figures 5, 6; Harper et al., 1998, 2000; Henderson et al., 2002; Macey et al., 2003b, 2006; Macefield et al., 2006).

### Cerebellar contributions

The cerebellum, by virtue of its essential role in mediating vestibular action and somatomotor coordination, exerts major influences on blood pressure control. The regulatory actions of the cerebellar vermis were described over 60 years ago by Moruzzi (1948), and the close interaction with head movement and vestibular challenges by a series of animal studies which demonstrate the coordination roles for autonomic and somatomotor action, interactions with somatic activity to dampen blood pressure (Miura and Reis, 1969; Achari and Downman, 1970; Hockman et al., 1970; Lutherer et al., 1983, 1989; Reis and Golanov, 1997), and the close coupling of blood pressure with breathing mediated through the ventral medulla and cerebellum (Harper et al., 1999; Rector et al., 2006). The essential role for the cerebellum in hypotension has been further demonstrated by animal neuroimaging in early feline life (Henderson et al., 2004). The developmental studies in the feline model demonstrate the substantial reorganization in control of blood pressure in early life, before rostral structures have adequately developed, and while projecting axons are missing myelin; after day 20–24 in the kitten, a remarkable switch from cerebellar to forebrain activation to hypotension emerges (Gozal et al., 1995a). Reorganization of blood pressure challenges can also be observed in responses of the ventral medullary surface through optical imaging procedures (Gozal et al., 1995a). That reorganization has significant implications for infants, who go through a critical period of risk for sudden infant death syndrome; there is little risk early in life, a high risk period from 2 to 4 months, followed by low risk. Impaired switching of blood pressure regulatory system organization during the risk period has the potential for fatal consequences until the pathways are fully developed.

**Figure 8:**
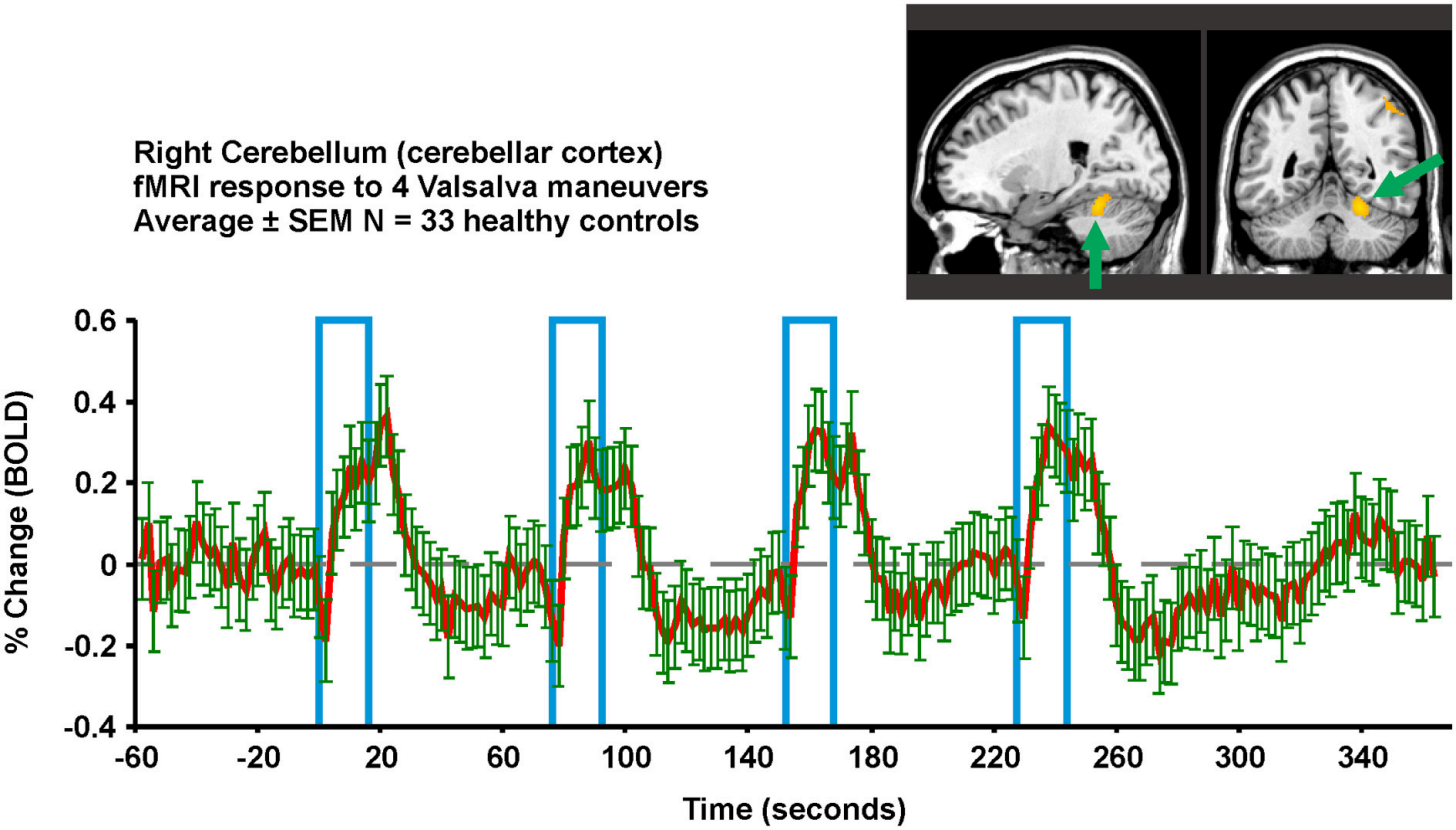
**Valsalva fMRI responses in the right superior cerebellar cortex in 33 healthy controls (23 male, age mean ± sd [range] = 52.3 ± 7.7 years)**. Region-of-interest from which signal is extracted is overlaid in yellow on an anatomical background. The protocol consists of four 18 s challenges of 30 mmHg minimum expiratory pressure, 1 min apart. All subjects achieved this pressure for all four challenges. Red is mean and green error bars are SEM (Data from Ogren et al., 2012).

Multiple fMRI findings confirm the involvement of the cerebellum in humans during autonomic challenges (Figures 5, 6, 8). The cerebellar cortex responds regionally to blood pressure changes, including respiratory loading (Gozal et al., 1995b; Macey et al., 2003b), lower body negative pressure (Kimmerly et al., 2007), Valsalva (Harper et al., 2000; Henderson et al., 2002), cold pressor (Richardson et al., 2013), end-expiratory breath-hold and Mueller maneuver (Kimmerly et al., 2013), and static hand grip (Macefield and Henderson, 2015). Figure 8 illustrates the time-course of cerebellar responses to a series of four Valsalva maneuvers; the signal increases rapidly, then plateaus during the expiratory period, and then rises rapidly to a peak 2–3 time-points wide (4–6 s), before falling below baseline and gradually recovering. This pattern does not follow a boxcar shape as in Figure 1, and therefore alternative analysis methods, such as time-trend analyses are better suited to detecting the sequence of neural responses to such an autonomic challenge. The cerebellar data are consistent with a dampening or coordinating role for the cerebellum in the presence of significant changes in blood pressure, which could similar to the motor coordination role traditionally associated with the structure (Thach et al., 1992).

### Cortical contributions other than limbic regions

Autonomic challenges used in fMRI studies lead to activation in other sensorimotor cortical areas, but these changes may relate to the task rather than autonomic control *per se*. For example, the hand grip requires visual attention to a signal, voluntary motor action, and varying sensory input from the hand; the task therefore leads to changes in the visual, motor, and sensory cortices in the hand-representation regions independent of autonomic regulation-related changes, as shown in Figure 6 (large yellow “blobs” principally on the left side). Regions which are considered integral to central autonomic regulation include the insula, VMPFC, and mid-cingulate, and can also be seen in Figure 6. However, these cortical areas do not show consistent activation across challenges, suggesting lateral prefrontal, parietal, and temporal activations found during some challenges are specific to those tasks. For example, whereas hand grip challenges lead predominantly to VMPFC deactivation (Figure 6; Goswami et al., 2011, 2012; Norton et al., 2013), the lateral prefrontal cortex shows strong activation to the Valsalva (Henderson et al., 2002). Few tasks show occipital activation, even though visual cues are used. Appropriate interpretation of fMRI findings will include both autonomic and challenge-related function.

### Lateralization

Autonomic functions in the brain are lateralized (Harper et al., 2000), in a manner reminiscent of other functions including motor, sensory, and language systems. The cold pressor and hand grip challenges show multiple structures with lateralized responses to the challenges, notably the mid, and posterior insula (Figures 5, 6). In adolescents, the amygdala, hippocampus, and ventral cerebellum show opposite responses on the left and right sides to a Valsalva maneuver (Ogren et al., 2010). The insular cortex is of particular interest with respect to lateralized autonomic function, as the left side function appears to be preferentially parasympathetic and the right side preferentially sympathetic (Oppenheimer and Cechetto, 1990; Oppenheimer et al., 1996); in two case studies, resection of the left insula led to minimal autonomic changes, but resection of the right led to less sympathetic and more parasympathetic activity (De Morree et al., 2015). In support of this left-right distinction, we showed that the right insula consistently shows greater activation than the left during the sympathetic phase of a Valsalva (Figure 7; Macey et al., 2012). The lateralization of function has obvious implications for stroke or other injury, since unilateral damage would impact the extent and timing of blood pressure regulation.

### Sex differences

Neuroimaging has demonstrated substantial sex differences in autonomic regulation (Kimmerly et al., 2007; Wong et al., 2007a). These neural differences are likely related to the observed differences in peripheral autonomic functions, including blood pressure regulation, sympathetic, and cardiovascular reactivity (Macey et al., 2013). While most of the imaging studies of autonomic function to date have presented findings in mixed sex groups, those that have separated responses by sex have invariably found magnitude and pattern differences between females and males. While this topic is deserving of a separate review, the influence of sex is mentioned here since it is a factor that should be considered in any autonomic neuroimaging study.

### Summary of findings in healthy people

Autonomic challenges elicit brain activation detectable by fMRI in healthy adults and children. The findings confirm evidence of networks shown in animal and earlier human stimulation and stroke studies, and have also demonstrated involvement of previously unrecognized brain regions, and functional relationships between separate regions. The neuroimaging findings demonstrate that autonomic action is a network phenomenon, with responses involving multiple, lateralized systems working together in a dynamic manner over time.

Measurement in healthy people can serve as a norm for identifying altered function in diseased conditions. Issues of experimental design, analytic procedures, and sensitivity of MRI protocols are current challenges to direct comparisons between experiments. However, studies using case-control designs allow for investigation of altered function.

## Types of pathology detectable by fMRI: findings in obstructive sleep apnea, heart failure, and congenital central hypoventilation syndrome

The types of neural alteration that can be detected by fMRI of autonomic actions goes beyond simply identifying more or less activation in a patient group, in contrast with other commonly-used fMRI stimuli. Our group has performed studies of autonomic function using neuroimaging in sleep-disordered breathing conditions, notably obstructive sleep apnea (OSA) and congenital central hypoventilation syndrome (CCHS; Harper et al., 2015), as well as heart failure (HF), which is often accompanied by central and obstructive sleep apnea (Henderson et al., 2002, 2003; Harper et al., 2003, 2005; Macey et al., 2003b, 2004a, 2005a,b,c, 2006; Woo et al., 2005, 2007; Serber et al., 2007; Ogren et al., 2010, 2012). Our studies used case-control, cross sectional designs to highlight areas of differences relative to healthy controls in the patient neural responses to standard autonomic stimuli. One unavoidable potential confound of comparisons between healthy and disease conditions is that the fMRI BOLD response may be influenced by the pathological state of the brain. Severe hypercapnia leads to delayed and reduced magnitude of BOLD responses (Cohen et al., 2002), so conditions of associated with high PaCO_2_ would likely lead to systematically altered fMRI responses. Hematocrit levels show moderate associations with BOLD responses (Levin et al., 2001), so variations associated with disease state or medication usage may influence findings. However, the general pattern of change is not altered in disease states, even following major brain insult such as stroke (Pineiro et al., 2002), so fMRI comparisons between healthy people and patients without major injury should be valid, at least for differences with moderate effect sizes. In conditions of sleep-disordered breathing without history of brain injury or disease (CCHS, HF, and OSA), differences appeared in which brain areas were recruited and in the patterns of responses of particular brain regions. Patient populations frequently showing dampened, delayed, or different responses compared with healthy controls; this section briefly describes three ways in which neural responses can differ.

### Magnitude-altered responses

Most brain structures that show differences in responses in people with sleep-disordered breathing exhibit a lower magnitude of change relative to patterns seen in healthy people. Early studies of the Valsalva in OSA illustrated much weaker responses in areas of the brainstem (dorsal pons, dorsal medulla), parietal cortex (left inferior), cingulate, temporal gyrus, and especially regions in the cerebellum (Henderson et al., 2003). Presumably, these dampened brain responses are linked with the dampened and phase-shifted heart rate responses to the same autonomic challenges (Macey et al., 2013). The cerebellum is affected in CCHS, showing greatly reduced responses relative to controls to the Valsalva, in contrast with the hippocampus which shows higher patient activation (Ogren et al., 2010). What cannot be distinguished with standard fMRI is whether the people with CCHS or OSA are starting at the same level of baseline activity as healthy controls. Since resting cerebral blood flow differs in OSA (Yadav et al., 2013; Baril et al., 2015), it is possible that this flow represents different tonic resting activity, and thus the responsiveness could be limited by a ceiling effect (Macey, 2015). Alternatively, if the baseline state is very low in OSA, the same change in fMRI signal may represent a relatively larger activation; that said, this possibility is less likely as the fMRI signal is calculated as a percent change from the baseline. Exaggerated responses in patient groups are also found in some regions in response to particular challenges, notably in brainstem regions of CCHS patients during the Valsalva (Ogren et al., 2010).

### Time-altered responses

Delayed responses to challenges are common in people with sleep disordered breathing. The delay in neural responses matches the delay in peripheral heart rate responses to autonomic challenges (Macey et al., 2013). Shorter challenges, such as the Valsalva or a brief hand grip, are well-suited to detecting delays, as can be seen by the detailed patterns evident in the timetrend of a Valsalva response in controls (Figure 8), or the delays in CCHS in the brainstem (Ogren et al., 2010), and in HF in the right insula (Ogren et al., 2012). These delays could arise from slower processing in a particular region, or from delayed inputs from sensory or other brain regions. The slower processing likely puts the people at risk of not adapting blood pressure or heart rate sufficiently fast to maintain optimum perfusion for some situations. However, CCHS, HF, and OSA patients also show a subset of structures with phase-leading signals, suggesting higher sensitivity in some regions in those conditions; such timing differences may lead to asymmetric sympathetic outflow, which would enhance the potential for fatal arrhythmias, a major consideration in sudden death in infants and epilepsy (Scorza et al., 2008, 2011; Harper and Kinney, 2010). Both the hippocampus and amygdala show neuronal discharge related to the cardiac and respiratory cycle in patients with epilepsy (Frysinger and Harper, 1989, 1990), an issue of concern when structural injury appears in such patients (Staba et al., 2007; Ogren et al., 2009a,b), and sudden death in epilepsy (SUDEP) very likely develops from a failure in breathing or the cardiovascular system (Nashef et al., 2007).

### Pattern-altered responses

Altered responses in disease conditions sometimes include different patterns as opposed to different amplitudes or latencies of the healthy pattern. The previously-mentioned delayed and dampened responses characteristic of many brain regions nevertheless follow the same pattern as in healthy people—the shape of the time course is similar. However, a small number of brain areas show patterns so different that in some cases they are opposite, including decreasing in contrast to increases in healthy controls, or the opposite, increasing in contrast to control decreases. Examples of divergent responses in OSA include the right anterior insula to expiratory loading (Macey et al., 2003b), the left anterior insular cortex to inspiratory loading (Macey et al., 2006), and the dorsal medulla and ventral thalamus to forehead cold pressor (Harper et al., 2003). The insula shows a signal decline to the Valsalva in healthy controls, but no change or a slight increase in CCHS (Ogren et al., 2010). Such differences are striking and may reflect a different functional organization in the disease condition, as opposed to an impairment (slower, weaker) of the normal function.

A further pattern apparent in challenges with recovery periods lasting tens of seconds or minutes is a more-rapid return to baseline. The Valsalva, in particular, highlights a weak undershoot and a rapid return to baseline in OSA patients after the strain period (Henderson et al., 2003). Since the undershoot is reflective of healthy cardiovascular physiology (Kalbfleisch et al., 1978; Porth et al., 1984), the weak fMRI undershoot and rapid return to baseline suggest impaired central autonomic regulation lead to the dampened and delayed cardiovascular changes in OSA (Macey et al., 2013).

## New technologies and future investigations

### Global bold signal: an indicator of total cerebral blood volume and oxygenation

**Figure 9:**
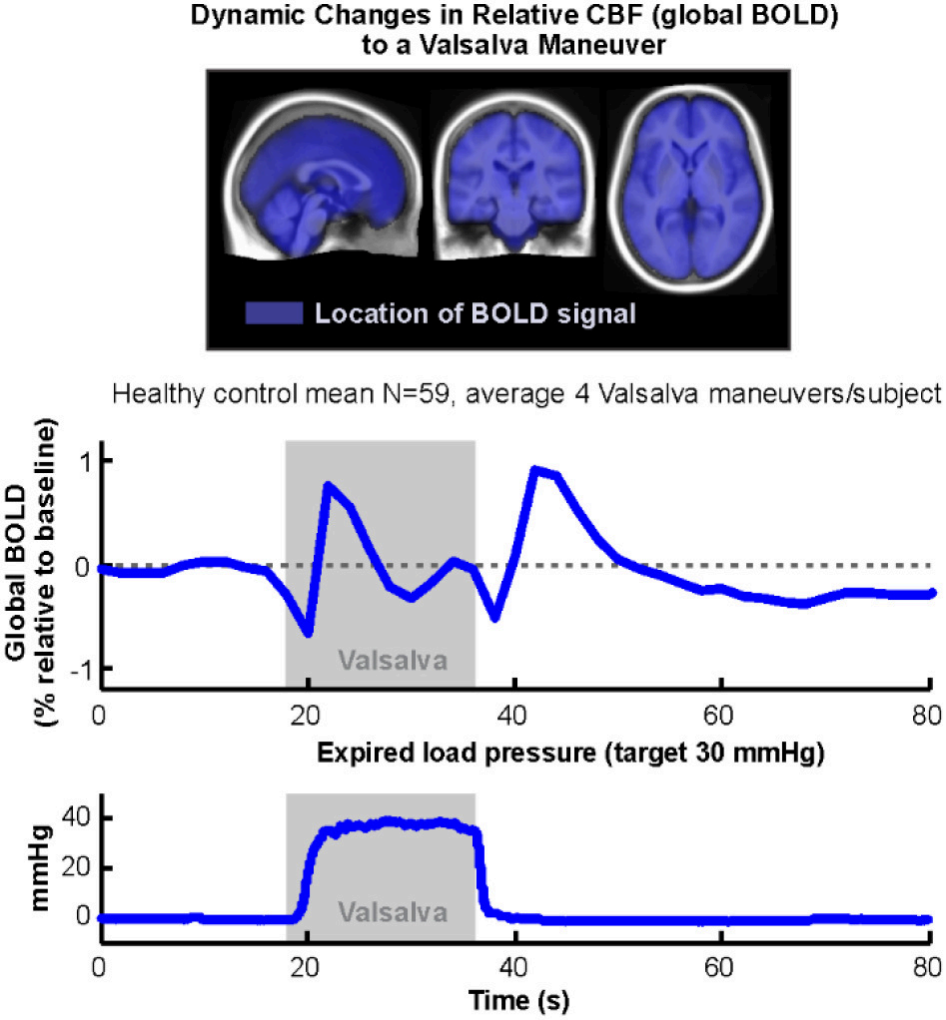
**Global BOLD signal during an average of four Valsalva challenges averaged over 59 healthy subjects (18 s duration, target pressure 30 mmHg, starting at time 18 s)**. Mean of expiratory pressures in bottom graph. All subjects achieved target pressure for all four challenges. Blue overlays on average anatomical background indicate locations from which global BOLD signal was calculated (Data from Macey et al., 2014).

Functional MRI measures the BOLD signal, which is sensitive to regional variations in blood volume and oxygenation, and which is usually assumed to reflect neural activation. In addition, however, vascular changes due to overall cerebral autoregulation lead to overall blood volume changes, which are effectively independent of neural activation-related changes (Kim et al., 1999), and thus, are usually excluded as confounds from fMRI analyses (Macey et al., 2004b). However, the global BOLD signal, that is the average of all in-brain voxels (Figure 9, top), is closely associated with these overall vascular effects, and can be used as an indirect measure of relative changes in cerebral blood volume and oxygenation. We have demonstrated differences in OSA during autonomic challenges reflective of weaker cerebrovascular responses, which presumably could place the patients at risk for brief periods of inadequate tissue perfusion (Figure 9; Macey et al., 2014). We also found large global BOLD differences in children with CCHS in response to gas challenges (Macey et al., 2003a). The global BOLD measurement is available with any BOLD-fMRI series, and offers an opportunity to measure a unique variable independent of neural activation.

### Cerebral blood flow and other measures with arterial spin labeling

Arterial spin labeling (ASL) is a non-invasive, MRI-based technique that quantifies cerebral blood flow (CBF). The method can be used to quantify *resting* CBF, as we have performed in OSA (Yadav et al., 2013) and CCHS (Macey et al., 2010), but of interest to autonomic function is the capacity to quantify dynamic changes (Wang et al., 2005). The advantage of quantitative techniques in neuroimaging is that absolute values of tissue or physiological characteristics can be measured and compared across people, and across MRI scanners, and over time in the same person. Positron emission tomography is also quantitative, but ASL has the advantage of being non-invasive and allows for continuous measurements over time. Normative values for quantitative measures can be established, which provides for comparison of a person or group with a norm, as opposed to a control group. Relative techniques, such as fMRI, require a control group assessed under the same conditions and protocol as a patient group. Arterial spin labeling is especially appealing for assessment of minutes-long autonomic challenges, because, in contrast with fMRI, the signal is stable over time so either longer protocols can be used, or a baseline could be followed with a separate scan many minutes later. For similar measurement stability reasons, the technique is amenable to a single task protocol, and might be preferable for a >2 min cold pressor challenge, for example.

A promising extension of ASL is measuring the permeability of the blood brain barrier (Gregori et al., 2013); while this method is still in development, the capacity to measure this variable non-invasively could provide unique insights into autonomic function and consequences of dysfunction, as with early indications in OSA of impaired permeability (Palomares et al., 2015).

### Measuring the effective transverse relaxation rate (R2^*^) to separate blood volume from blood oxygenation

The MRI parameter effective transverse relaxation rate (R2^*^), or 1/T26^*^, is sensitive to brain tissue oxygenation, and can be acquired in a similar manner to conventional BOLD fMRI images (Glover et al., 1996). The measure is based on the difference in signal intensity between two fMRI images collected at different echo times (TE1 and TE2; Bohr et al., 2015, used 13 and 40 ms). A standard fMRI sequence collects images at a single echo time (for example, 30 ms in Ogren et al., 2012). The shorter echo time (TE1) is less sensitive to oxygenation than the longer echo time (TE2), so the change in intensity at TE1 reflects cerebral blood volume, and at TE2 is reflective of the combination of cerebral blood volume and oxygenation. In the example in Bohr et al., the end of Phase II of the Valsalva sees a cerebral-blood volume reduction-induced decline in both TE1 and TE2, but a greater decline in TE2 due to the reduced oxygenation at that time (Bohr et al., 2015). While a standard fMRI sequence is sensitive to changes in both blood volume and oxygenation, and is termed a T2^*^ sensitive contrast, the single echo time does not allow separation of the two influences or quantification of T2^*^. The dual echo R2^*^ sequence allows for a quantitative measure calculated from the intensity difference between the two echo time images. This measure is especially applicable to autonomic challenges, where changes in oxygenation are common. Although the technique was proposed some time ago (Moonen et al., 1994), there are few studies taking advantage of this measure. In one such recent study, the functional brain changes elicited by the Valsalva maneuver were separated into blood volume and oxygenation components, demonstrating that deoxygenation began 10 s into the challenge (Bohr et al., 2015). Longer challenges involving breath manipulation would be likely to benefit from measuring T2^*^ rather than the conventional BOLD contrast images.

### Increased spatial and time resolution with multiband scanning protocols

Multiband imaging is a development in MRI acquisition design for acquiring multiple slices of data in a single pass, and thus can accelerate multislice sequences. The approach can improve signal-to-noise, temporal, and spatial resolution (Moeller et al., 2010). State-of-the-art multiband sequences available on standard 3 Tesla MRI scanners using standard manufacturer-provided head coils allow for a remarkable increase in time resolution to sub-second levels (0.7 s or even 0.5 s; Blaimer et al., 2013; Tomasi et al., 2015); this sampling interval is close to the maximum needed to resolve the hemodynamic response function. However, the high time resolution also allows for temporal brain dynamics to be investigated with respect to one region preceding or following activation of a reference region. Moving from 0.5 to nearly two samples per second acquisition offers the possibility of resolving latencies and time delays, critical aspects of understanding systems with feedback, hysteresis, and error correction, as are present in autonomic regulation. Also remarkable is that this six-fold increase in temporal resolution is accompanied by an increase in spatial resolution to 2 × 2 × 2 mm, a six-fold increase from a typical 3.5 × 3.5 × 4 mm. Greater spatial resolution can be obtained (1.5 × 1.5 × 1.5 mm) at the expense of slightly longer acquisition times. The six-fold increase in spatial resolution allows for smaller regions to be investigated, which may be especially important for assessing autonomic function in brainstem and diencephalic areas.

### Functional diffusion imaging

Diffusion weighted fMRI (dw-fMRI) is a technique under development that closely tracks neural activity with the potential for greatly enhanced temporal and spatial resolution compared to BOLD fMRI. While the basis of fMRI is the correlation between the hemodynamic response and local activation, which is diffuse in time and spatial extent, a more direct neural activity correlate is neuronal size changes with activation (Lux et al., 1986). This phenomenon depends on fluid dynamics during cell firing that modify cell size, and results in variation in reflected light as an indication of neural activity (Lipton, 1973; Rector and Harper, 1991; Andrew and MacVicar, 1994). Proof-of-concept studies confirm that dw-fMRI is sensitive to changes in activation (Tsurugizawa et al., 2013), and tracks BOLD activations to a standard memory task (Aso et al., 2013). While standard diffusion technologies allow 125 ms acquisition times for a 3 × 3 × 4 mm voxel size (TR 1000 ms, eight slices in Aso et al., 2013), the previously-mentioned multiband protocols enable 35 ms acquisition times for a 1.5 × 1.5 × 1.5 mm voxel size (Tomasi et al., 2015), so dw-fMRI has the potential to bring much-needed improvements in timing resolution to autonomic studies.

### Magnetic resonance spectroscopy

Magnetic resonance spectroscopy (MRS) measures levels of brain chemicals such as metabolites, structural cellular molecules, and neurotransmitters. Traditional MRS, termed one-dimensional MRS, is restricted to detecting 3–5 metabolites (Frahm et al., 1989), whereas other approaches including spectral editing and especially two-dimensional MRS allow detection of γ-aminobutyric acid (GABA), glutamate, lactate, and 15–20 other chemicals (Keltner et al., 1996; Thomas et al., 2001). Since neurotransmitters and some metabolites are state-dependent, 2D-MRS could be applicable to autonomic challenges with effects lasting 30–60 min or longer.

### Other analytic methodologies

The time course of responses to autonomic challenges is suited to modeling with a dynamic systems approach. While fMRI methodology has included structural equation modeling (Kim et al., 2007), this method does not address the dynamic component, that is, consideration of the timing of responses across regions. Dynamic causal modeling (DCM), as implemented in the SPM software package, assesses neural responses as a system of related activations influencing each other, with consideration for time delays (Friston et al., 2003), and this approach offers the opportunity to extent autonomic imaging analyses. The DCM method may be more suited to protocols involving repeated tasks, as opposed to single challenges. Perhaps the greatest limiting factor in the use of DCM is that possible circuitry needs to be defined beforehand. However, the potential benefit of DCM or other dynamic system analyses is that highly complex systems can be simplified, and pathology could potentially be narrowed to a smaller number of brain regions, in the case where some brain regions are functioning normally at the local level, but receiving different inputs, and hence showing different activation patterns.

Model-free approaches allow identification of patterns and regions without a priori definitions, as with VOI or whole-brain SPM analyses. Interparticipant correlations (Hejnar et al., 2007), independent components analysis (Calhoun et al., 2001), and finite impulse response functions (Kay et al., 2008) are examples of methods that allow detection of patterns of activation across subjects without specifying a model or region. However, such methods often produce findings that are difficult to interpret, such as activation patterns during baseline periods. The model-free approaches need careful application, and are likely suitable to only a subset of autonomic neuroimaging studies.

Multi-modality imaging is now commonplace, but analytic approaches that account for multiple measures remain limited. Most fMRI studies performed currently also involve collecting anatomical, resting state, and DTI data, and ASL measures of cerebral blood flow may at some point also become standard. Such measures are typically analyzed in parallel, with separate findings for each modality. The current limitations on multi-modality analyses are statistical and signal processing in nature, rather than inadequate computing power or storage space. Multi-modality approaches from other fields, such as statistics and engineering, have been adapted to disease classification problems (Hinrichs et al., 2009; Xu et al., 2015), and offer the opportunity to extend our scientific understanding of central autonomic control.

## Conclusions

Neuroimaging has confirmed previous findings of involvement of multiple brain structures other than the brainstem in autonomic regulation. Furthermore, the importance of limbic brain regions and the cerebellum has been demonstrated with such studies, and the concept of a central autonomic network has significantly expanded from initially proposed regions. Functional MRI studies, with high temporal resolution, have shown the time course of responses of key modulatory brain areas, such as the insula, cerebellum, and cingulate (to name a few), from which the timing of neural responses has been identified. Activity in these sites appears in conditions of sleep disordered breathing and heart failure to be often dampened and time-shifted, which will impair autonomic functions. Recent scanning and analytic methodologies offer the opportunity to investigate human autonomic control in more depth, including in disease conditions where insights will ultimately guide new, effective interventions.

## Author contributions

RH, RK, and PM contributed to design, data collection, analysis, and interpretation of original findings underlying the content of this review. JO contributed to analysis and interpretation of original findings. RH, JO, and PM contributed to initial drafting of manuscript. All authors contributed to writing revising, and approving the manuscript, and are accountable for all aspects of the work.

### Conflict of interest statement

The authors declare that the research was conducted in the absence of any commercial or financial relationships that could be construed as a potential conflict of interest. The reviewer Jennifer A. Ogren and handling Editor declared a current collaboration and the handling Editor states that the process nevertheless met the standards of a fair and objective review.
